# The effectiveness of a standardized tobacco cessation program on psychophysiological parameters in patients with addiction undergoing long-term rehabilitation: a quasi-experimental pilot study

**DOI:** 10.1186/s12916-024-03405-z

**Published:** 2024-05-01

**Authors:** J. Fuchshuber, H. Schöber, M. Wohlmuth, H. Senra, C. Rominger, A. Schwerdtfeger, H. F. Unterrainer

**Affiliations:** 1 Department of Psychoanalysis and Psychotherapy, Medical University Vienna, Vienna, Austria; 2 Center for Integrative Addiction Research (CIAR), Grüner Kreis Society, Vienna, Austria; 3 Institute of Psychology, University of Graz, Graz, Austria; 4 IEETA, University of Aveiro, Aveiro, Portugal; 5 School of Health and Social Care, University of Essex, Colchester, UK; 6 University Clinic for Psychiatry and Psychotherapeutic Medicine, Medical University Graz, Graz, Austria; 7 Department of Religious Studies, University of Vienna, Vienna, Austria; 8 Faculty of Psychotherapy Science, Sigmund Freud University, Vienna, Austria

**Keywords:** Tobacco cessation, Tobacco dependence, Addiction therapy outcome, Heart rate variability, Self-efficacy

## Abstract

**Background:**

Although there is a very high comorbidity between tobacco dependence and other addictive disorders, there are only few studies examining the implementation and outcomes of a tobacco cessation program in patients with addictive diseases. Therefore, the aim of this study is to investigate to what extent a standardized tobacco cessation program leads to improvements regarding psychological/physical parameters in patients with addiction undergoing therapy and whether there is a reduction in tobacco consumption.

**Methods:**

The study took place in a therapeutic community specialized in addiction therapy. A total sample of 56 participants were non-randomly assigned to an intervention group (IG; *n* = 31) and a treatment as usual group (TAUG; *n* = 25). The IG participated in a 6-week tobacco cessation program, while the TAUG received no additional treatment. Both groups were assessed for changes in primary outcomes (tobacco dependence, smoked cigarettes per day (CPD), and general substance-related craving) and secondary outcomes (heart rate variability (HRV): root mean square of successive differences, self-efficacy, and comorbid psychiatric symptoms) at two measurement time points (pre- and post-treatment/6 weeks).

**Results:**

We observed significant improvements in self-efficacy (*F*_(1,53)_ = 5.86; *p* < .05; *η*_*p*_^2^ = .11) and decreased CPD in the IG (*β* = 1.16, *ρ* < .05), while no significant changes were observed in the TAUG. No significant interaction effects were observed in psychiatric symptoms, general substance-related craving, and HRV.

**Conclusions:**

The results highlight the potential benefit of an additional tobacco cessation program as part of a general addiction treatment. Although no improvements in the physiological domain were observed, there were significant improvements regarding self-efficacy and CPD in the IG compared to the TAUG. Randomized controlled trials on larger samples would be an important next step.

**Trial registration:**

ISRCTN15684371

## Background

Tobacco dependence is the most widespread addictive disorder worldwide. Although tobacco use is associated with a wide range of systemic diseases and is indirectly responsible for about 20% of deaths within developed countries [1] and 15% of deaths specifically in Austria [2], about 22% of women and 27% of men describe themselves as daily smokers. Notably, the prevalence (> 65%) of tobacco addiction among patients with substance use disorder receiving inpatient treatment is strikingly high [3, 4]. With 22.4% comorbid disorders, the general tobacco-dependent population shows a degree of comorbidity that almost doubles the rate of the non-dependent one [5]. Recent research even suggests, that genetic liability for smoking intensity and tobacco dependence may play a causal role in the development of mental disorders [6].

Individuals who smoke and have concomitant psychiatric comorbidities are at increased risk in several respects. Not only do they suffer increased physical morbidity and mortality, but they also have a poorer prognosis in terms of comorbid psychiatric symptoms [7]. In addition, the co-occurrence of smoking, other addictive behaviors, and mental health disorders can complicate treatment and affect both psychiatric treatment outcomes and tobacco cessation efforts. Although the concept of providing safe and competent cessation treatment in psychiatric settings is fundamentally sound, it is rarely implemented in practice [8].

Especially in regard to the treatment of patients suffering from severe forms of addiction disorders related to opioids and polytoxicomania, smoking is often seen as a relatively less harmful behavioral alternative. In this context, treatment providers are often wary of the effects of tobacco cessation for their patients. Common fears include an increased risk of relapse to other substances or the acceleration of underlying mental health conditions like depression [9, 10]. Furthermore, after a successful inpatient detoxification, many patients start to attend self-help meetings such as Narcotics Anonymous to maintain abstinence. In these meetings, smoking is usually allowed or even encouraged, while it may be warned to not make too many additional changes, which would include the cessation of tobacco use [8, 11, 12]. What is more, personnel in drug abuse treatment facilities often smoke themselves, which further increases resistance to the implementation of smoking cessation programs in such institutions [13, 14]. Empirically, most clinical studies on the effects of smoking cessation in patients suffering from other addictions indicate either positive or a least neutral consequences for the general addiction recovery process [8], with only one report highlighting negative effects on the treatment of alcoholism [15]. Hence, additional provision of standardized and evidence-based tobacco cessation programs in clinics could lead, e.g., to improved self-efficacy, which in turn might contribute to improved effectiveness of overall addiction treatment [8, 16].

However, to this date, no previous study investigated the effects of a tobacco cessation program within a therapeutic community specialized for the treatment of opioid addiction and poly drug use. The specific therapeutic community approach in addiction treatment emphasizes an integrative recovery- and attachment-oriented approach which can be summarized by the phrase “community as method” (see [17] for a detailed description). Previous research in therapeutic communities suggests a very high proportion (95%) of patients diagnosed with opioid dependence exhibit a comorbid tobacco addiction [18].

At the physiological level, diminished heart rate variability (HRV) has previously been associated with a wide range of substance use disorders [19–21], encompassing acute and chronic consequences of addiction to tobacco smoking. HRV, defined as the fluctuation in heart rate around the mean, offers a noninvasive method for assessing autonomic functions. In general terms, elevated HRV signifies heightened parasympathetic as well as decreased sympathetic activation [22]. It is linked to several aspects of physical and mental health including more adaptive emotion regulation and higher well-being [23]. One way in which smoking negatively impacts cardiovascular function is by influencing the control of the autonomic nervous system [24]. Successful tobacco cessation interventions have been consistently associated with increased HRV [25–28] underscoring the substantial health benefits associated with tobacco abstinence.

### Research aims

This pilot study examines the potential benefit of a standardized tobacco cessation program in a group of patients undergoing long-term addiction treatment. An intervention group (IG; participants in the program) and a control group receiving treatment as usual group (TAUG; non-participants in the program) in an inpatient therapeutic community are examined for changes in primary outcomes (tobacco dependence, smoked cigarettes per day (CPD) and general substance-related craving) and secondary outcomes (HRV, self-efficacy, and comorbid psychiatric symptoms).

## Methods

### Participants and procedure

A written, standardized consent form was signed by each person. The inclusion criteria were voluntary participation, a current tobacco dependence (F17.2), the desire to quit smoking, and an age > 18 years. No other in- or exclusion criteria were applied. The study was approved by the ethics committee of Karl-Franzens-University Graz (GZ. 39/30/63 ex 2022/23). The psychological measures were assessed before and after the tobacco cessation program (1.5 weeks before and after the first/last session, 9 weeks apart from each other). The collection of sociodemographic variables included sex, age, comorbidity (diagnosed by a licensed psychiatrist), and current medication. Operationalized outcome measures were tobacco dependence, CPD, general substance-related craving, self-efficacy, general psychiatric symptom severity, and HRV.

All patients were diagnosed with poly-drug use disorder (F19.2) by a licensed psychiatrist according to the International Classification of Diseases version 10 [29]. Pre-treatment, the consumption pattern of all participants was characterized by a chaotic use of psychoactive substances, including almost all substance classes (e.g., opioids, tranquilizers, stimulants, alcohol, cannabinoids, and tobacco) with opioids being the primary drug of abuse in all participants. The pretreatment substance use was assessed via medical-case-history data assessed by the psychiatrist of the therapeutic community.

The recruitment and examination took place at the addiction clinic Schloss Johnsdorf of the Grüner Kreis Society. The standard treatment within this therapeutic community — which all participants received — consists of group therapy (once a week), individual psychotherapy (once a week), counseling by social workers, psychiatric consultations as well as sport, art, and work therapy.

With regard to the specifically vulnerable population studied, the present pilot study design was a quasi-experimental controlled trial, without random assignment. While all participants explicitly expressed the wish to stop smoking, this procedure enabled the consideration of patients’ concerns regarding potential negative impacts on their drug addiction recovery. None of the participants took part in previous smoking cessation programs. No additional smoking cessation medication was implemented in the study.

A-priori sample size calculations were performed with G-Power (version 3.1.9.6), for a mixed-design ANOVA with one interaction between group (2 groups) and assessments (2 repeated measurements). Considering the lack of prior evidence on the intervention effectiveness, the mixed-design ANOVA was set for a medium effect size (Cohen’s *F* = 0.25), a statistical Power (1-β) of 0.9, a α of 0.5, an intra-correlation coefficient of 0.5 and nonsphericity correction of 1, resulting in a minimum sample size of 46 subjects, and a critical *F* = 4.06.

The “smoke free in 6 weeks” program is a standardized behavioral therapy intervention (see [30] for detailed instructions) that takes place once a week for 1.5 h over 6 weeks and is provided by the Austrian health insurance provider *Österreichische Gesundheitskasse* (ÖGK). Held in the therapeutic community, it employed behavioral therapy coupled with personalized recommendations for nicotine replacement. The Austrian Health Insurance standard for therapy guided the program’s content, which was tailored for inpatient clients. Specifically, the following interventions and cognitive-behavioral therapy techniques were applied in the weekly group sessions: Psychoeducation including information on tobacco addiction, health risks, advice on relapse prevention and the handling of craving, motivation building, behavioral observation, setting of the personal quit-smoking day, behavior modification through the development of alternative actions, training of progressive muscle relaxation, strengthening of self-efficacy and information on the topics of physical activity and nutrition. Finally, to increase intrinsic motivation, each session included an analysis using calibrated carbon monoxide measurements. The primary aim was to help clients achieve smoking cessation, favoring this approach over gradual reduction methods. To support ongoing abstinence and motivation, participants were directed to additional resources such as the Smoke-Free App [31], Smoke-Free Phone [32], and regional outpatient cessation services. This program was led by a clinical psychologist.

### Psychometric assessment

#### Psychiatric symptoms

The BSI-18 [33] is a shortened version of the Brief Symptom Inventory which consists of 18 items rated on a 5-point Likert scale. The instrument exhibited an excellent internal consistency ranging from McDonalds *ω*_(t1)_ = 0.90 to *ω*_(t2)_ = 0.93 and captured self-rated symptoms of psychiatric distress during the last seven days. The sub-scales include somatization, depressiveness, and anxiety. In addition, a total Global Severity Index (GSI) score of psychiatric symptom severity can be generated by summing up the sub-scales.

#### Tobacco dependence

The Fagerström Test for Cigarette Dependence (formerly Fragerström Test for Nicotine Dependence; FTND; [34]) is a self-assessment instrument measuring tobacco dependence intensity [35]. It consists of 6 items that assess the amount of smoked cigarettes per day (CPD), compulsive use, and dependence intensity. The test is composed of yes/no items and multiple choice questions, leading to a total score with a good retest-reliability of 0.88 [36]. Internal consistency in the present study ranged from *ω*_(t1)_ = 0.67 to *ω*_(t2)_ = 0.73.

#### General substance-related craving

The MaCS (Mannheim Craving Scale; [37]) is an instrument for measuring substance-related craving. It consists of 12 items and four additional items and is rated on a 5-point Likert scale. Internal consistency ranged between *ω*_(t1)_ = 0.89 and *ω*_(t2)_ = 0.93.

#### Self-efficacy

The self-efficacy scale (SWE; [38]) is a self-report instrument for measuring general optimistic self-confidence. It consists of 10 items and measures optimistic competence expectancy, i.e., confidence in solving difficult situations with one’s own abilities, on a 5-point Likert scale. Internal consistency for the total scale ranged from *ω*_(t1)_ = 0.82 to *ω*_(t2)_ = 0.79.

### Physiological assessment

#### Heart rate variability

The HRV was measured with electrocardiography (ECG). ECG is a procedure that measures the electrical activity of the heart muscle fibers. During the heartbeat, the heart muscles change their electrical properties, which can be measured with electrodes. HRV was recorded by portable ECG once before and once after the intervention via HRV short-time measurement (5 min), assessed with a three-channel electrocardiograph (Varioport-Becker Meditec, Karlsruhe). The measurement took place in a quiet environment in the morning and the patients were asked not to move and to adopt a relaxed posture. HRV parameters were analyzed offline with Kubios premium software vers. 3.5 [39], applying artifact correction if necessary. In order to keep the alpha-error inflation to a minimum, only the root mean square of successive differences (RMSSD) was investigated, as it is the most commonly used metric in studies measuring HRV [40]. Moreover, it is relatively sensitive to short-term changes, stable and reliable in short-time measurements, and a well-documented indicator for stress responses of the autonomic nervous system [41–43]. For the analysis, the RMSSD was logistically transformed in order to ensure a normal distribution.

### Statistical analysis

Data management, *t* tests, chi^2^ tests, Fisher exact tests, Friedman tests, Kolmogorov–Smirnov tests, Levene tests, and repeated measures ANOVA (2 × 2 design: group × time) were performed using the SPSS 29. The alpha level was set to 0.05 (two-tailed). Post hoc analysis for multiple comparisons was undertaken using the Bonferroni correction, in order to investigate group × time interaction effects. A repeated measures proportional odds logistic regression model using a generalized estimating equation (GEE) was undertaken to investigate the potential effect of the intervention for reducing the number of CPD (ordinal variable). The GEE analysis was computed using the repolr R package, using robust variance estimators. Due to incomplete data, the analyses required the following exclusions: one TAUG patient from the self-efficacy analysis, one patient each from IG and TAUG in the GSI analysis, and for HRV data, one IG and four TAUG patients.

## Results

### Sample and descriptive statistics

The total sample consisted of 56 participants (age = 18–61; female: *n* = 8; TAUG: *n* = 25; IG: *n* = 31). As detailed in Table 1, the most frequent comorbid disorders in the IG were affective disorders (29%), while neurotic stress-related and somatoform disorders dominated in the TAUG (20%). Moreover, besides maintenance medication (IG: 32%; TAUG: 28%), antidepressants were most frequently prescribed in the IG (28%), in contrast to neuroleptics in the TAUG (20%). No other medications were prescribed during the cessation intervention. In both groups, cannabis was the most frequently abused substance before the start of their treatment (IG: 94%; TAUG: 88%). The majority of participants consumed opioids intravenously before the treatment (IG: 87%; TAUG: 84%). Furthermore, most patients smoked 11–20 CPD at baseline (IG: 42%; TAU: 56%). Generally, no significant differences within the outcome measures were observed at baseline (all *p* > 0.05).

**Table 1:** Characteristics of the study participants

Variable	Intervention group (*n* = 31)	TAU group (*n* = 25)	*p*
Female	5 (17%)	3 (14%)	ns
Male	26 (84%)	22 (88%)	
Age: mean (SD)	32y (12y)	31y (10y)	ns
ICD diagnosis			
Comorbidity	18 (58.1%)	12 (48%)	ns
F20–F29 schizophrenia; schizotypal and delusional disorders	5 (16%)	4 (16%)	
F30–F39 affective disorders	9 (29%)	3 (12%)	
F40–F48 neurotic stress-related and somatoform disorders	8 (28%)	5 (20%)	
F50–F59 behavioral syndromes associated with physiological disturbances and physical factors	1 (3%)	0 (0%)	
F60–F69 personality and behavioral disorders	4 (13%)	1 (4%)	
Medication			ns
Anticonvulsants	3 (10%)	4 (16%)	
Antidepressants	9 (28%)	4 (16%)	
Maintenance	10 (32%)	7 (28%)	
Neuroleptics	6 (19%)	5 (20%)	
Pretreatment substance abuse or dependence			ns
Alcohol	19 (61%)	16 (64%)	
Opioids	31 (100%)	25 (100%)	
Cannabis	29 (94%)	22 (88%)	
Sedatives, hypnotics, anxiolytics	16 (52%)	12 (48%)	
Cocaine	22 (71%)	18 (72%)	
Other stimulants, excluding caffeine	13 (42%)	11 (44%)	
Hallucinogens	11 (35%)	9 (36%)	
Nicotine	31 (100%)	25 (100%)	
Pretreatment intravenous consumption	27 (87%)	21 (84%)	ns
Smoked cigarettes per day:			ns
< 10	3 (10%)	0 (0%)	
11–20	13 (42%)	14 (56%)	
21–30	12 (39%)	8 (32%)	
> 31	3 (10%)	3 (12%)	
Tobacco dependence, baseline mean (SD)	5.53 (2.09)	6.27 (3.07)	ns
Self-efficacy (SD)	2.75 (.33)	2.81 (.43)	ns
Craving, baseline mean (SD)	1.29 (0.54)	1.53 (.73)	ns
Psychiatric symptoms, baseline mean (SD)	.84 (.59)	.89 (73)	ns
HRV (RMSSD), baseline mean (SD)	3.23 (.62)	3.09 (.74)	ns

*p*-column indicates significance level regarding either *t*, chi^2^, or Fisher exact tests

*HRV* heart rate variability, *RMSSD* root mean square of successive differences, RMSSD was logistically transformed

### Outcome

Kolmogorov–Smirnov tests indicated that all dependent variables analyzed via ANOVA (tobacco dependence, general substance-related craving, self-efficacy, general psychiatric symptom severity, and HRV) were normally distributed (all *p* > 0.05). Levene tests indicated homogeneity of variance for all of these variables (all *p* > 0.05).

As shown in Table 2, a significant interaction effect was observed regarding self-efficacy (*F*_(1,53)_ = 5.86; *p* < 0.05; *η*_*p*_^2^ = 0.11). Moreover, the interaction time × group was significantly associated with the number of CPD (*β* = 1.16, *ρ* < 0.05). In the repolr R package results, positive coefficients should be interpreted as suggesting a tendency for the ordinal score (dependent variable) to decrease, and a negative score as a tendency for the ordinal score to increase [44]. The positive regression coefficient seen for the interaction, therefore, suggests a significant decrease in the number of CPD for patients in the intervention group who completed the treatment (end-of-treatment stage), in comparison with patients in the control group (at the end-of-treatment stage).

**Table 2:** Means, standard deviations, within effects, and interaction effects of group (IG vs. TAUG) × time (T1 vs. T2)

Variable			**Mean values and standard deviations**				**Within effect (t1 vs t2; ANOVA)**				**Group × time interaction** **(2 × 2 ANOVA)**			
			*M*(t1)	SD(t1)	*M*(t2)	SD(t2)	*F*	df	*p*	*η*_*p*_^2^	*F*	df	*p*	*η*_*p*_^2^
Self-efficacy	TAUG		2.81	.43	2.79	.45	3.80	1.53	.06	-	5.86	1.53	.02	.10
	IG		2.75	.49	2.95	.33								
Psychiatric symptoms	TAUG		.91	.74	.91	.80	.31	1.52	.58	-	1.83	1.52	.67	-
	IG		.82	.60	.76	.63								
Craving	TAUG		1.53	.73	1.30	.54	22.16	1.54	.00	.29	1.88	1.54	.18	-
	IG		1.29	.60	.88	.53								
Tobacco dependence	TAUG		6.20	1.76	6.00	1.73	3.67	1.54	.06	-	.96	1.54	.33	-
	IG		5.53	2.03	4.91	2.31								
HRV (RMSSD)	TAUG		3.09	.74	3.24	.73	1.48	1.47	.23	-	.51	1.47	.48	-
	IG		3.23	.62	3.23	.60								
			Frequencies and percentage								Group × time interaction (GEE)			
		Category	*n*(t1)	%(t1)	*n*(t2)	%(t2)					Wald-*χ*^2^	df	*p*	*B*
Smoked cigarettes per day	TAUG	< 10 11–20 21–30 > 31	0 14 8 3	0% 56% 32% 12%	0 13 11 1	0% 52% 44% 4%					4.72	1	.03	1.16
	IG	< 10 11–20 21–30 > 31	3 13 12 3	10% 42% 39% 10%	8 15 5 3	26% 48% 16% 10%								

*TAUG* treatment as usual group, *IG* intervention group, *HRV* heart rate variability, *RMSSD* root mean square of successive differences, RMSSD was logistically transformed, *GEE* generalized estimating equations, *p* < .05

No interaction effects were found regarding changes in total tobacco dependence, craving, HRV or psychiatric symptom severity (all *p* > 0.05). Bonferroni corrected post hoc tests revealed a significant increase in self-efficacy in IG (*M*_(t1)_ = 2.75, SD_(t1)_ = 0.49, *M*_(t2)_ = 2.95, SD_(t2)_ = 0.33; *F*_(1,53)_ = 10.94, *p* < 0.01, *η*_*p*_^2^ = 0.18) but not in TAUG (*M*_(t1)_ = 2.81, SD_(t1)_ = 0.49, *M*_(t2)_ = 2.79, SD_(t2)_ = 0.45; *p* > 0.05). Similarly, we observed an decrease in IG regarding total tobacco dependence (*M*_(t1)_ = 5.53, SD_(t1)_ = 2.03, *M*_(t2)_ = 4.91, SD_(t2)_ = 2.31; *F*_(1,54)_ = 4.80, *p* < 0.05, *η*_*p*_^2^ = 0.08) but not in TAUG (*M*_(t1)_ = 6.20, SD_(t1)_ = 1.76, *M*_(t2)_ = 6.00, SD_(t2)_ = 1.73; *p* > 0.05). The frequency of participants in the IG who smoked < 10 CPD increased form *n*_(t1)_ = 3 (10%), to *n*_(t2)_ = 8 (26%), while the number of patients who smoked between 21 and 30 CPD decreased from *n*_(t1)_ = 12 (39%) to *n*_(t2)_ = 5 (16%).

Finally, both groups exhibited a significant decrease in general substance-related craving (*F*_(1,54)_ = 22.15; *p* < 0.001; *η*_*p*_^2^ = 0.29). Bonferroni corrected post hoc analyses indicated a decrease in craving for the IG corresponding to *F*_(1,54)_ = 20.68 (*M*_(t1)_ = 1.29, SD_(t1)_ = 0.60, *M*_(t2)_ = 0.88, SD_(t2)_ = 0.53; *p* < 0.001; *η*_*p*_^2^ = 0.28), while the TAUG exhibited a decrease of *F*_(1,54)_ = 5.03 (*M*_(t1)_ = 1.53, SD_(t1)_ = 0.73, *M*_(t2)_ = 1.30, SD_(t2)_ = 0.54; *p* < 0.05; *η*_*p*_^2^ = 0.09).

## Discussion

In this pilot study, we examined the potential benefit of a standardized tobacco cessation program among patients undergoing treatment for a diagnosed addictive disorder at an inpatient facility. Our results provide preliminary insights into the effectiveness of the intervention in enhancing self-efficacy. Furthermore, while failing to significantly impact HRV or total tobacco dependence, in contrast to the TAUG, the IG showed a significant decrease in CPD. Finally, both groups showed a significant decrease in general substance-related craving, hinting towards the effect of the inpatient therapeutic community treatment.

Considering the limited sample size, power, and non-randomization of our pilot study, the results should be interpreted with caution [45]. Specifically, the non-randomized design of the study might have led to a situation in which both patient groups wished to stop smoking and did not significantly differ in their tobacco dependence in baseline, but the IG might have been more motivated to do so than the TAUG. In turn, this might lead to an overestimation of our observed effects. However, our preliminary but promising findings can be seen as a building block for further research using a randomized control design. Smoking cessation for patients suffering from severe addiction disorders is often discussed controversially, with patients and self-help groups, but also clinical professionals expressing concerns regarding possible detrimental effects of such interventions [8, 46]. Hence, empirical research on and practical implementation of such procedures are often met with considerable resistance in naturalistic settings. In correspondence to this, the presented findings indicate modest but overall positive effects of smoking cessation in the specific setting of a therapeutic community and might contribute to facilitating further research in this area.

Nevertheless, as previous studies investigating cognitive-behaviorally oriented approaches [47], indicated moderate effectiveness in reducing tobacco dependence, our results highlight the specific difficulties of such an intervention with regard to an inpatient facility treating patients diagnosed with severe addiction disorders. Furthermore, it is important to note that due to a lack of follow-up data, the sustainability of these effects over the long term remains uncertain at this point.

To address these concerns discussed above and minimize potential outcome bias, future investigations must implement randomizing assignments to intervention and control groups together with at least one point of follow-up measurement. This approach will allow for a more robust assessment of the durable impact of the program and contribute to our understanding of its effectiveness over time. Subsequent studies should, therefore, consider more conservative effect sizes than those observed in our study with regard to sample size calculations.

Furthermore, gender-specific aspects of tobacco cessation should be addressed, as previous research indicated that smoking cessation interventions show generally less success in women [48].

Of note, both groups showed a significant decrease in the general substance-related craving. This finding resonates well with previous studies investigating the effectiveness of addiction treatment in therapeutic communities [49, 50].

## Conclusions

The results of this pilot study suggest the promising efficacy of the evaluated standardized smoking cessation approach in regard to the improvement of self-efficacy and CPD in a population of patients in recovery from addictive disorders linked to opioids and polysubstance dependence. Considering the existing limitations that restrict interpretability, future studies using a randomized controlled design and including follow-up measurements are warranted.

## Data Availability

Data and materials will be made available by request to the corresponding author.

## Abbreviations

CPD: Cigarettes per day
ECG: Electrocardiography
FTND: Fagerström Test for Cigarette Dependence
GEE: Generalized estimating equation
HRV: Heart rate variability
IG: Intervention group
MaCS: Mannheim Craving Scale
ÖGK: Österreichische Gesundheitskasse
RMSSD: Root mean square of successive differences
SWE: Self-efficacy scale
TAUG: Treatment as usual group
